# Genome-Wide Association Study Identifies Candidate Genes for Body Size Traits in Wanyue Black Pigs

**DOI:** 10.3390/ani16010117

**Published:** 2025-12-31

**Authors:** Haibo Ye, Wei Li, Fang Tian, Qianqian Wang, Zhonghua Ma, Jinyu Guan, Yueyun Ding, Xianrui Zheng, Zongjun Yin, Xiaodong Zhang

**Affiliations:** 1 College of Animal Science and Technology, Anhui Agricultural University, Hefei 230036, China; yhb183264796560823@163.com (H.Y.); liweirfi@126.com (W.L.); 18391007746@163.com (F.T.); 15656098967@163.com (Q.W.); 13140669641@163.com (Z.M.); guanjy324@163.com (J.G.); dingyueyun@ahau.edu.cn (Y.D.); zxr07sk1@163.com (X.Z.); yinzongjun@ahau.edu.cn (Z.Y.); 2 Anhui Province Key Laboratory of Local Livestock and Poultry Genetic Resource Conservation and Bio-Breeding, Anhui Agricultural University, Hefei 230036, China

**Keywords:** body size traits, genome-wide association study, single nucleotide polymorphism, pig

## Abstract

This study aimed to elucidate the genetic basis of body-size-related traits in Wanyue Black pigs, including body length, chest circumference, forearm circumference, and hip circumference. Phenotypic data were collected from 139 four-month-old female pigs, and genotyping was performed using a 50K SNP array. After stringent quality control and genotype imputation, approximately 4,697,453 high-quality autosomal variants were retained for subsequent genome-wide association study (GWAS), transcriptome-wide association study (TWAS), and phenome-wide association study (PheWAS). The GWAS identified four genome-wide significant loci, including rs343276492 and rs321308815. TWAS results revealed that the expression level of PTH2R was significantly associated with pituitary-related traits. In addition, selection signal analysis identified multiple genomic regions related to growth, development, and environmental adaptability, which were significantly enriched in pathways such as circadian rhythm regulation and the MAPK signaling pathway. These pathways play critical roles in growth regulation and adaptive evolution in Wanyue Black pigs. Collectively, this study provides valuable candidate genes and potential molecular markers for the genetic improvement and breeding of Wanyue Black pigs.

## Simple Summary

This study focuses on the body size traits such as body length and chest girth of the Wanyue Black Pig, aiming to explore its genetic basis to assist in breed improvement. The study involved genotyping and phenotype data collection from 139 sows. Using various genetic analysis methods, multiple genetic loci and candidate genes related to body size were identified, and selection signals associated with growth and adaptability were also detected. These findings reveal the genetic regulatory mechanisms underlying the body size traits of the Wanyue Black Pig and provide practical molecular markers. These markers can help improve the breeding efficiency of this local pig breed and are of significant importance for ensuring pork supply and promoting the development of the pig industry.

## 1. Introduction

Pork is one of the most important sources of animal protein for humans [1]. In China in particular, pork consumption has consistently ranked first, playing a critical role in both dietary structure and national food security [2]. With the continuous development of the swine industry, enhancing growth performance and production efficiency has become a central objective of breeding programs. In this context, body measurements have received considerable attention as key indicators reflecting body conformation and growth development in pigs [3]. Commonly assessed body measurements include body length (BL), chest circumference (CC), shank circumference (SC), and leg–hip circumference (LHC) [4,5]. Body size traits generally exhibit moderate to high heritability and are therefore regarded as important targets for molecular breeding and genetic improvement. A study using automated posture estimation technology analyzed the genetic parameters of body size traits such as hip width and shoulder width in pigs, and the results indicated that these traits had moderate to high heritability [6]. Similarly, genome-wide association studies in Duroc and Landrace × Yorkshire crossbred pigs revealed that the heritability estimates for most body size traits also ranged from moderate to high [7]. These findings suggest that body size traits hold great potential for application in genetic improvement programs. Notably, body size traits not only reflect an individual’s conformation and growth performance but are also closely associated with a variety of economically important traits, including carcass characteristics, reproductive capacity, and feed conversion efficiency [8,9]. Therefore, investigating body size traits is of great importance for elucidating their genetic basis and advancing molecular breeding and genetic improvement in pigs. Body size traits are often characterized by strong genetic correlations. For instance, the genetic correlation between chest circumference and abdominal circumference can reach 0.747 [10], while that between chest width and hip width is as high as 0.840 [11]. These findings suggest that body size traits may share common regulatory mechanisms at the genetic level. Building on this characteristic, we employed the multivariate linear mixed model (mvLMM) implemented in GEMMA for genome-wide association analysis, with the aim of leveraging the genetic correlations among traits to enhance detection power and achieve a more comprehensive identification of candidate genes.

With the rapid advancement of molecular genetics, GWAS has become an indispensable tool for dissecting the genetic architecture of complex traits [12]. In pigs, GWAS has been widely applied to uncover the genetic basis of body size and carcass traits. However, the candidate genes identified vary across different pig breeds. For example, a GWAS of 439 Landrace × Yorkshire crossbred pigs on the number of thoracolumbar vertebrae (NTLV) and ribs (NR) identified four significant loci, including rs3469762345, and revealed candidate genes such as *ALDH7A1*, *PTPRT*, and *PAK1*, which have been demonstrated to play key roles in skeletal development [13]. In Qinchuan Black pigs, GWAS of 10 growth traits identified 18 candidate genes associated with muscle and developmental processes, with *TENM3* considered a potential regulator of growth traits in this breed [14]. In a study of 358 Dongliao Black pigs, GWAS of body weight and average daily gain revealed 39 QTLs significantly associated with these traits and identified six candidate genes, including *MACROD2* [15]. Furthermore, GWAS of 1405 body mass index (BMI) records from 924 Yunnan Black pigs annotated 29 protein-coding genes, among which *FABP2*, a key gene for human BMI, was significantly enriched in fatty acid binding and lipid digestion and absorption pathways [16]. A study investigating the genetic mechanisms underlying pH variations measured in the longissimus dorsi muscle of Beijing Black pigs at 2 h and 24 h postmortem identified *SYT5* and *SNX13* as key candidate genes influencing pH at both time points. These findings enhance our understanding of the genetic factors affecting pork quality and safety, and provide insights for improving meat quality through genetic selection [17]. Collectively, these studies indicate that although breed-specific GWAS reveal distinct candidate genes, most of them are closely related to skeletal development, muscle growth, and metabolic processes, providing important insights into the genetic mechanisms underlying body size traits in pigs. Nevertheless, research on Wanyue Black pigs remains limited, and the genetic basis of their body size traits requires further investigation.

This study investigated 139 Wanyue Black pigs, a high-quality local breed from Anhui Province characterized by strong reproductive capacity, tender meat, and abundant intramuscular fat [18]. Based on 50K liquid-phase chip genotyping data, missing genotypes were imputed using BEAGLE, and genome-wide association studies were performed with GEMMA through both single-marker and multi-marker models. This strategy systematically identified genetic variants associated with body size traits. The primary aim was to elucidate the genetic regulatory mechanisms underlying these traits in Wanyue Black pigs, thereby providing a theoretical foundation and molecular marker resources to support molecular breeding and genetic improvement of the breed.

## 2. Materials and Methods

### 2.1. Ethics Approval

All animals used in this study were handled in accordance with the guidelines for the care and use of laboratory animals established by the Ministry of Agriculture of China. Tissue samples were collected from pigs after obtaining approval from the Ethics Committee of Anhui Agricultural University (approval number: AHAU20221113).

### 2.2. Experimental Animals

This study used 139 four-month-old Wanyue Black pigs, obtained from Huimingshan Co., Ltd. (Anqing, China). All pigs were maintained under standardized environmental conditions and remained healthy throughout the study period. Body measurements were taken with pigs in a natural standing position. Each animal was gently restrained by trained personnel, and measurements were recorded on-site using a flexible tape measure and specialized instruments. Specifically, measurements were defined as follows: BL was the straight-line distance from the midpoint of the upper edge of the left ear root to the midpoint of the upper edge of the right ear root and then to the base of the tail; CC was measured as the vertical circumference of the thorax behind the scapula; SC was the circumference at the narrowest point of the left forelimb; and LHC was the distance from the anterior edge of the left knee, passing the anus, to the anterior edge of the right knee. Descriptive statistics for all traits, including sample size, maximum, minimum, mean (±standard deviation), and coefficient of variation, were calculated using R software (R version 4.4.0) [19]. To investigate genetic correlations among the traits, Pearson correlation coefficients were calculated for the four measurements. Correlation analyses were performed using the cor() function in R, with significance set at *p* < 0.01.

### 2.3. Genotyping, Imputation, and Quality Control

Ear tissue samples were collected from 139 Wanyue Black pigs, and genomic DNA was extracted using a Tiangen Genomic DNA Extraction Kit (Tiangen Biotech Co., Ltd., Beijing, China). DNA concentration and quality were assessed using a NanoDrop 2000 spectrophotometer (Thermo Fisher Scientific, Wilmington, DE, USA) in combination with 1.5% agarose gel electrophoresis. Prior to genotyping, all DNA samples were diluted to a final concentration of 50 ng/μL.

Genotyping was performed using a customized porcine 50K SNP liquid chip. Raw genotyping data were subjected to quality control based on the following criteria: individual call rate ≥ 90%, SNP call rate ≥ 90%, minor allele frequency (MAF) ≥ 0.01, and exclusion of SNPs located on sex chromosomes. Genotype imputation was conducted using the porcine haplotype reference panel PHARP v4.0, which comprises 6449 individuals from 154 pig breeds and populations worldwide and is based on the Sus scrofa genome assembly Sscrofa11.1. This reference panel includes approximately 56 million SNPs and insertion–deletion variants (indels), as well as ~223,000 structural variants. Prior to imputation, conform-gt software (Version 2.0) was used to assess strand alignment and allele compatibility between the target and reference panels [20]. Haplotype pre-phasing and genotype imputation were then performed using Beagle v5.0 under default parameters.

After imputation, SNPs were further filtered based on an imputation quality threshold of R^2^ ≥ 0.9 and MAF ≥ 0.05, resulting in a total of 4,697,453 high-quality autosomal SNPs. This imputed SNP dataset was consistently used for all downstream analyses, including genome-wide association analysis, genome-wide significance threshold calculation, transcriptome-wide association study, and phenome-wide association study.

SNPs were grouped into predefined MAF intervals, and the proportion of variants within each interval was calculated using R software (R version 4.4.0). Statistical analyses were performed using functions from the stats package, including *t*-tests and non-parametric tests, while data manipulation was conducted using the dplyr package (verison 1.1.4) [21]. Data visualization was carried out using the ggplot2 package (verison 4.0.0) [22]. A significance threshold of *p* < 0.05 was applied throughout.

### 2.4. Genome-Wide Association Analysis of Body Size Traits

Genotyping was performed using a customized 50K SNP liquid chip, Data analysis indicated that SNPs were evenly distributed across all chromosomes. To further investigate the genetic basis of phenotypic variation, genome-wide association studies were conducted using GEMMA software (version 0.98.5) [23].

For four body size traits—body length (BL), chest circumference (CC), shank circumference (SC), and leg–hip circumference (LHC)—GWAS was conducted using GEMMA version 0.98.5 (https://github.com/genetics-statistics/GEMMA/releases, accessed on 15 October 2025) with a multivariate linear mixed model (mvLMM). In this model, genotypes were modeled as fixed effects, while additive polygenic effects were modeled as random effects. The statistical model was expressed as:

Y = WA + xβ^T^ + U + E; G ~ MN*_n_* × d(0, K, Vg), E ~ MN*_n_* × d(0, I*_n_*× *n*, Ve),

where Y is an *n* by d matrix of d phenotypes for n individuals; W = (w_1_, …, wc) is an *n* × c matrix of covariates (fixed effects) including a column of 1s; A is a c by d matrix of the corresponding coefficients including the intercept; x is an *n*-vector of marker genotypes; β is a d vector of marker effect sizes for the d phenotypes; U is an *n* by d matrix of random effects; E is an n by d matrix of errors; K is a known *n* × *n* relatedness matrix, I*_n_* × *n* is a *n* by *n* identity matrix, Vg is a d by d symmetric matrix of genetic variance component, Ve is a d by d symmetric matrix of environmental variance component and MN*_n_* × d(0, V_1_, V_2_) denotes the *n* × d matrix normal distribution with mean 0, row covariance matrix V_1_ (*n* by *n*), and column covariance matrix V_2_ (d by d).

GEMMA performs tests comparing the null hypothesis that the marker effect sizes for all phenotypes are zero, H_0_: β = 0, where 0 is a d-vector of zeros, against the general alternative H_1_: β ≠ 0. For each SNP in turn, GEMMA obtains either the maximum likelihood estimate (MLE) or the restricted maximum likelihood estimate (REML) of Vg and Ve, and outputs the corresponding *p* value. During multiple testing correction, recognizing that the conventional Bonferroni adjustment can be overly conservative, we incorporated a linkage disequilibrium (LD) pruning strategy to reduce the influence of redundant SNPs on association signals. The genome-wide significance threshold was set at 3.429 × 10^−7^.

Additionally, Manhattan and quantile–quantile (Q–Q) plots were generated using the R package CMplot (version 4.5.1), based on the latest pig reference genome assembly (Sscrofa11.1).

Linkage disequilibrium (LD) blocks within candidate regions were analyzed using Haploview 4.2 [24]. Genotype–phenotype associations were further illustrated by boxplots generated using the ggplot2 package (version 4.4.0) in R. The UCSC Genome Browser was used for SNP annotation [25]. SNPs located within genes were directly assigned to those genes, whereas SNPs outside genes were annotated to all genes within a 50 kb upstream and downstream window.

### 2.5. Transcriptome-Wide Association Study

Building on genetic variants associated with body size traits identified in previous genome-wide association studies in pigs, this study further conducted transcriptome-wide association studies (TWAS) to explore the role of gene expression in the genetic regulation of body size traits. TWAS analyses were performed using the FarmGTEx TWAS-Server (https://alphaindex.zju.edu.cn/animalTwas/index.php/run/twas.html) accessed on 15 October 2025 [26] an online platform that integrates genotype and transcriptome data to enable large-scale transcriptome-wide association analyses. During the analysis, SNP loci associated with body size traits identified by GWAS were integrated with transcriptome data from the entire pig genome. The transcriptome data were also obtained from the FarmGTEx database, including RNA-seq results across multiple pig tissues, and all datasets were standardized prior to analysis. Association analyses were conducted using the analytical tools provided by the FarmGTEx TWAS-Server.

### 2.6. Functional Annotation and Phenome-Wide Association Study

This study further performed functional annotation of the significant SNPs associated with pig body size traits. Phenome-Wide Association Study was conducted using the PigBiobank website (https://pigbiobank.farmgtex.org/) accessed on 15 October 2025, to explore the associations between these candidate genes and multiple economic traits in pigs. The PheWAS analysis relied on the online tools provided by PigBiobank, integrating pig genotype data with phenotypic data of various economic traits to identify significant genes related to body size traits.

### 2.7. Selective Sweep Analysis Between Wanyue Black and Huoshou Black Pigs

To systematically identify genomic regions under selection in Wanyue Black pigs (WYB) relative to their ancestral breed, the Hoshou Black pig (HS), a genome-wide selection signature analysis was performed based on whole-genome resequencing data. A total of 59 individuals were included in this analysis, comprising 29 HS pigs and 30 WYB pigs. Two complementary approaches were applied to detect selection signals across the genome. First, genetic differentiation between the two populations was estimated using the fixation index (F_ST) proposed by Weir and Cockerham, calculated with a sliding window of 50 kb and a step size of 25 kb. Concurrently, nucleotide diversity ratios (π_HS/π_WYB) were computed using the same window parameters to identify genomic regions exhibiting significantly reduced diversity in WYB, which may reflect selective sweeps occurring during domestication or breeding. Genomic windows ranking in the top 5% for both F_ST values (F_ST > 0.15) and nucleotide diversity ratios were defined as candidate regions under selection. Genes located within these candidate regions were annotated based on the pig reference genome (Sscrofa11.1), and Kyoto Encyclopedia of Genes and Genomes (KEGG) pathway enrichment analysis was conducted using the clusterProfiler package (version 4.2) in R in conjunction with the org.Ss.eg.db annotation database to elucidate the potential biological functions of the candidate genes [27].

## 3. Results

### 3.1. Statistical Characterization and Correlation Analysis of Phenotypic Traits

Descriptive statistics of body length (BL), chest circumference (CC), shank circumference (SC), and leg–hip circumference (LHC) of 139 samples are presented in Table 1. The mean (±standard deviation) of BL was 70.48 ± 9.51 cm with a coefficient of variation (CV) of 13.49%. The mean ± standard deviation of CC was 68.57 ± 8.08 cm, with a CV of 11.78%. The mean ± standard deviation of SC was 14.24 ± 2.03 cm, showing the highest CV (14.25%) among the four traits, while the mean ± standard deviation of LHC was 58.60 ± 4.96 cm, with the lowest CV (8.46%). Correlation analysis revealed extremely significant positive correlations (*p* < 0.01) between all trait pairs (Table 2). The correlation coefficients were 0.956 for BL and CC, 0.914 for BL and SC, 0.898 for BL and LHC, 0.935 for CC and SC, 0.893 for CC and LHC, and 0.873 for SC and LHC. These high correlations suggest that the four traits may be influenced by a common genetic regulatory mechanism.

### 3.2. Imputation Accuracy

**Figure 1:**
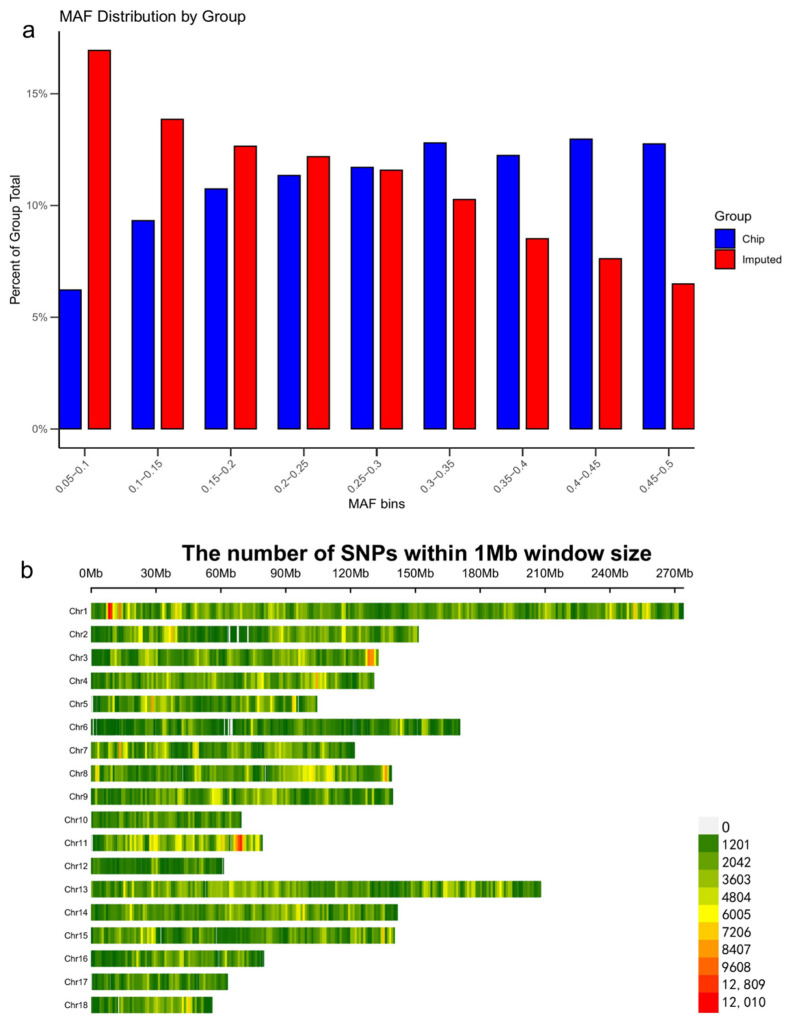
Minor Allele Frequency (MAF) Distribution of Chip and Imputed Datasets. (**a**) MAF Distribution by Group: Compares variant proportions across MAF bins for chip and imputed datasets; chip has high-frequency variants peaking at ~15% (0.45–0.51 MAF), imputed shows uniform, lower proportions (~5% in 0.41–0.51 MAF). (**b**) SNP Distribution Across Chromosomes: 4,697,453 SNPs (from 139 samples, 1 Mb window) uniformly distributed across 18 chromosomes.

Using minor allele frequency (MAF) distribution plots, we systematically compared the genetic variation patterns between the chip dataset and the imputed dataset across different MAF intervals, with the results as follows: Marked differences were observed in the distribution of variant frequencies between the two groups. The chip dataset was dominated by high-frequency variants, peaking within the MAF interval of 0.45–0.5, where variants accounted for ~15% of the total. In contrast, the imputed dataset displayed a relatively uniform distribution across all MAF intervals, with consistently lower proportions of variants. Specifically, the proportion of variants in the 0.41–0.51 and 0.42–0.51 intervals was only ~5%, substantially lower than in the chip dataset. Notably, in the low MAF intervals (e.g., 0.41–0.43), the frequency of rare variants in both groups approached zero, suggesting that the overall proportion of rare variants was low in both datasets (Figure 1a). Collectively, these findings indicate that the chip dataset was primarily composed of common, high-frequency variants, whereas the imputed dataset contained a more dispersed distribution of variants, with overall lower frequencies. In addition, genetic variant identification based on the genomic data of 139 samples yielded 4,697,453 single nucleotide polymorphisms (SNPs), which were uniformly distributed across all 18 chromosomes (Figure 1b).

### 3.3. Genome-Wide Association Analysis Identifies Candidate Loci and Genes Linked to Body-Size-Related Traits in Wanyue Black Pigs

**Figure 2:**
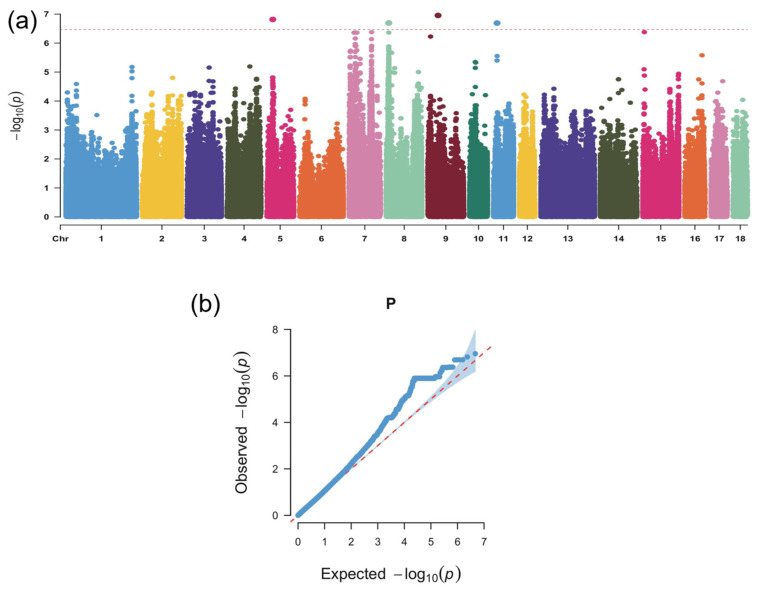
Genome-wide association analysis results. (**a**) Manhattan plot of the GWAS signals. The *x*-axis represents chromosome numbers (chromosomes 1–18), and the *y*-axis indicates −log_10_(*p*) values. The red horizontal line denotes the Bonferroni-corrected significance threshold (*p* < 3.429 × 10^−7^). A total of four SNPs reached genome-wide significance. (**b**) Quantile–quantile (Q–Q) plot for deviation assessment. The *x*-axis represents the expected −log_10_(*p*) values, and the *y*-axis represents the observed −log_10_(*p*) values. Most observed values closely follow the theoretical diagonal, indicating no evident population stratification or systematic bias and supporting the reliability of the GWAS results.

To further investigate the genetic basis of phenotypic variation, a genome-wide association analysis was performed using the GEMMA software (version 0.98.5). The Manhattan plot generated with the CmplotR package (verison 4.5.1) revealed a total of four SNPs exceeding the Bonferroni-corrected significance threshold (*p* < 3.429 × 10^−7^) (Figure 2a). These significant association loci exhibited a clear clustered distribution pattern across the chromosomes, suggesting the presence of genomic hotspot regions associated with the target traits. Evaluation of the Q–Q plot showed that most observed values closely followed the expected theoretical distribution along the diagonal, indicating no obvious population stratification or systematic bias and further supporting the reliability of the GWAS results (Figure 2b). Functional annotation of the significant loci identified six candidate genes, including *ALG9* and *COG6* (Table 3).

### 3.4. Linkage Disequilibrium and Genotypic Effect Analyses of Candidate SNPs Associated with Body Size Traits

**Figure 3:**
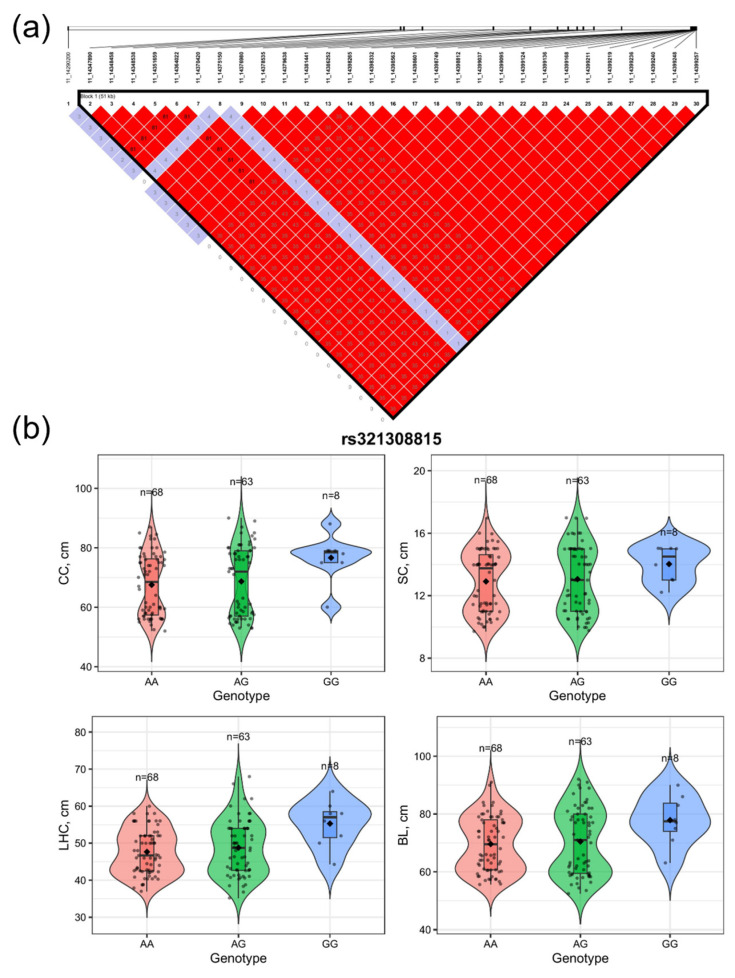
Linkage Disequilibrium and Genotypic Effects of SNP rs321308815 on Pig Body Size Traits. (**a**) LD analysis of rs321308815 region; reveals ~51 kb core LD block (Block 1) with higher LD coefficients in internal vs. external markers. (**b**) Genotypic effects of rs321308815 on pig body size traits (CC, SC, LHC, BL, cm; *n* = 63/68/8 for AA/AG/GG). GG has higher values in all traits.

Linkage disequilibrium (LD) analysis of the most significant locus, rs321308815, identified a core LD block spanning approximately 51 kb. The pairwise LD coefficients among markers within this block were markedly higher than those observed outside the block, indicating a strong local genetic linkage structure in the population (Figure 3a). Genotype effect analysis further revealed significant genotype-dependent differences in multiple body size traits, including chest circumference (CC), shoulder width (SC), leg–hip circumference (LHC), and body length (BL). Specifically, individuals carrying the GG genotype exhibited consistently higher phenotypic values for all four traits compared with those carrying the AA or AG genotypes (Figure 3b). Although the number of individuals with the GG genotype was relatively limited (*n* = 8), a consistent favorable trend was observed across all evaluated traits. Collectively, these results suggest that rs321308815 and its surrounding ~51 kb LD region may play a role in the genetic regulation of body size traits in pigs, although further validation in larger populations is required.

### 3.5. Transcriptome-Wide Association Study Reveals the Association Between Pituitary Traits and PTH2R Gene Expression

**Figure 4:**
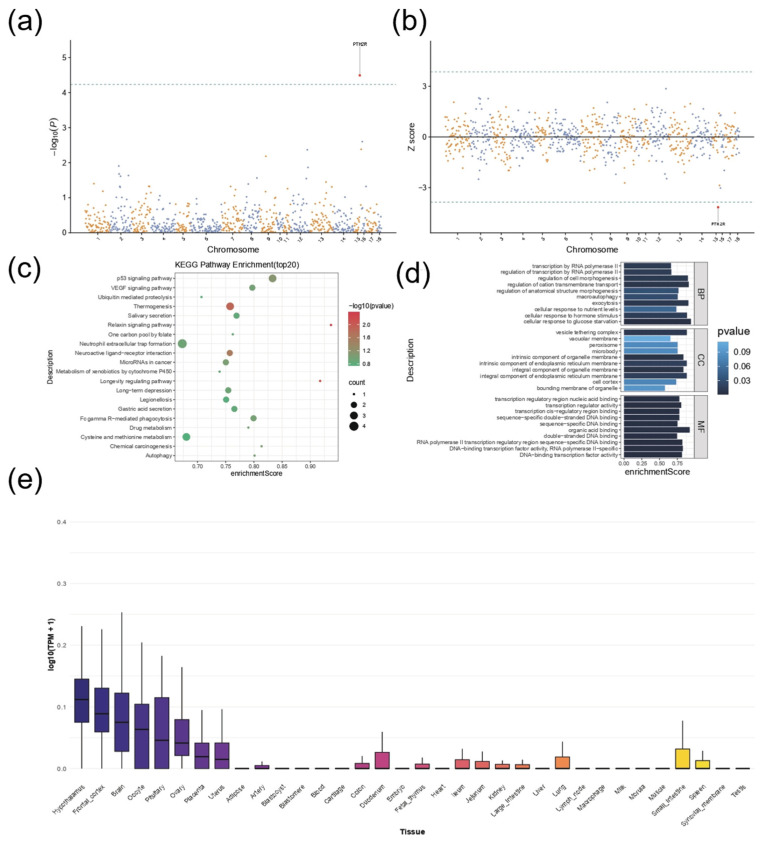
Genome-wide association study and transcriptome-wide association study results for pituitary-related traits. (**a**) Shows the result of integrating GWAS body size trait-associated genetic variants with genome-wide gene expression data; only pituitary traits exhibit significant variant–gene expression associations among tested tissue-specific traits. (**b**) Presents TWAS analysis results; PTH2R on chromosome 15 has a significant Z score, indicating its expression variation is closely linked to pituitary trait phenotypic variation. (**c**) Displays top 20 KEGG pathway enrichments; the p53 signaling pathway and VEGF signaling pathway show strong enrichment scores and significance. (**d**) Shows GO functional enrichment results (across biological process, cellular component, molecular function categories), including transcriptional regulation, organelle membrane, and DNA-binding transcription factor activity. (**e**) Presents bulk tissue gene expression profile of PTH2R (sorted by expression); PTH2R is highly expressed in neural system tissues (hypothalamus, cerebral cortex, brain) and reproductive endocrine-related tissues (oocytes, pituitary), with pituitary expression among the highest.

By integrating genetic variants associated with body size traits from GWAS with genome-wide gene expression data, we found that, among all tested tissue-specific traits, only the pituitary trait exhibited significant variant–gene expression associations (Figure 4a). Further TWAS analysis revealed that PTH2R on chromosome 15 showed a significant Z score, indicating that variation in its expression is closely associated with pituitary trait phenotypic variation (Figure 4b). Functional enrichment analysis further revealed potential molecular mechanisms: in KEGG pathways, the p53 signaling pathway and the VEGF signaling pathway showed particularly strong enrichment scores and significance (Figure 4c); GO analysis identified related functions across three categories, including transcriptional regulation and anatomical structure morphogenesis (biological process), organelle membrane and cell cortex (cellular component), and DNA-binding transcription factor activity (molecular function) (Figure 4d). In addition, tissue expression profiling of PTH2R showed markedly higher expression in neural system tissues such as the hypothalamus, cerebral cortex, and brain, as well as in reproductive-endocrine-related tissues such as oocytes and the pituitary, with pituitary expression consistently ranking among the highest (Figure 4e). Taken together, these results suggest that PTH2R may influence pituitary function and participate in molecular mechanisms such as the p53 signaling pathway and transcriptional regulation, thereby playing a critical role in the genetic regulation of body size traits in pigs.

### 3.6. Functional Annotation and Phenome-Wide Association Study Reveal Candidate Genes Associated with Body Size Traits in Pigs

**Figure 5:**
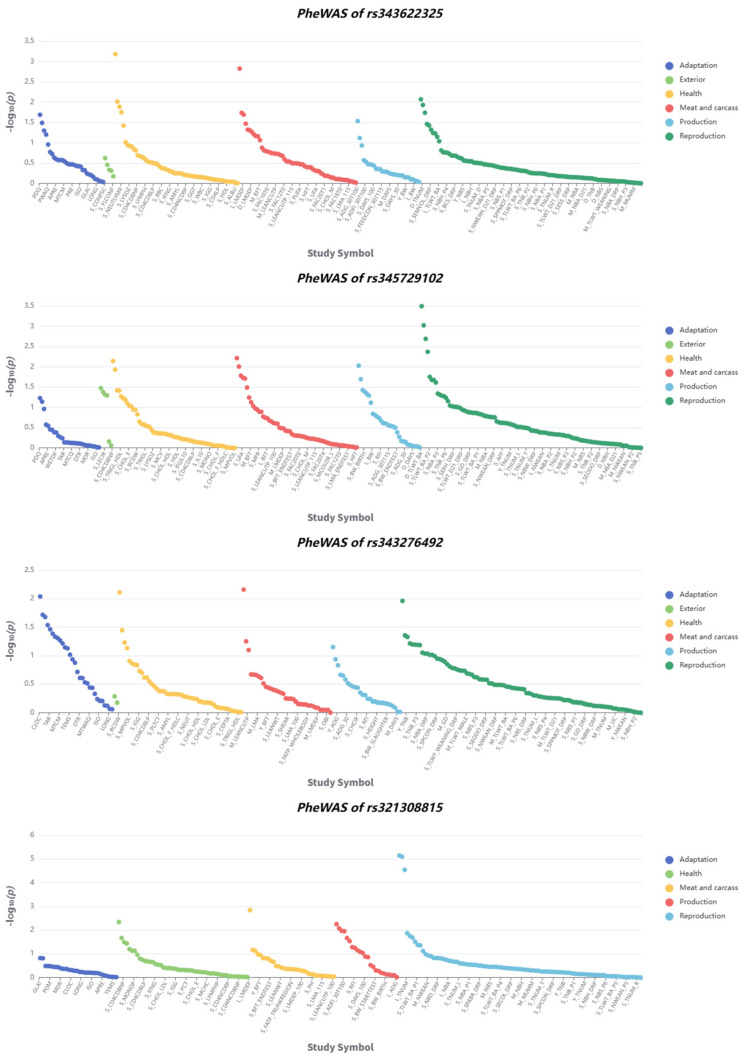
PheWAS results for four SNPs. This figure displays phenome-wide association study results for four SNPs (rs343622325, rs3435729102, rs343276492, rs321308815), with one subplot per SNP: Axes: *x*-axis = Study Symbol (corresponding to distinct phenotypes); *y*-axis = −log_10_(*p*) (association significance).

To further characterize the associations between the identified loci and phenotypic traits, a phenome-wide association study was conducted. The results revealed that all four loci were significantly associated with multiple economically important traits in pigs. Specifically, rs343622325 showed significant associations with both health-related and meat quality traits; rs321308815 and rs345729102 were primarily associated with reproduction-related phenotypes, whereas rs343276492 was significantly associated not only with meat quality and health-related traits but also with adaptability-related traits. Notably, rs321308815 shows association signals with −log_10_(*p*) > 5 for some phenotypes (Figure 5).

### 3.7. Genomic Selection Signals and Functional Pathway Analysis of Wanyue Black Pigs Relative to Huoshao Black Pigs

**Figure 6:**
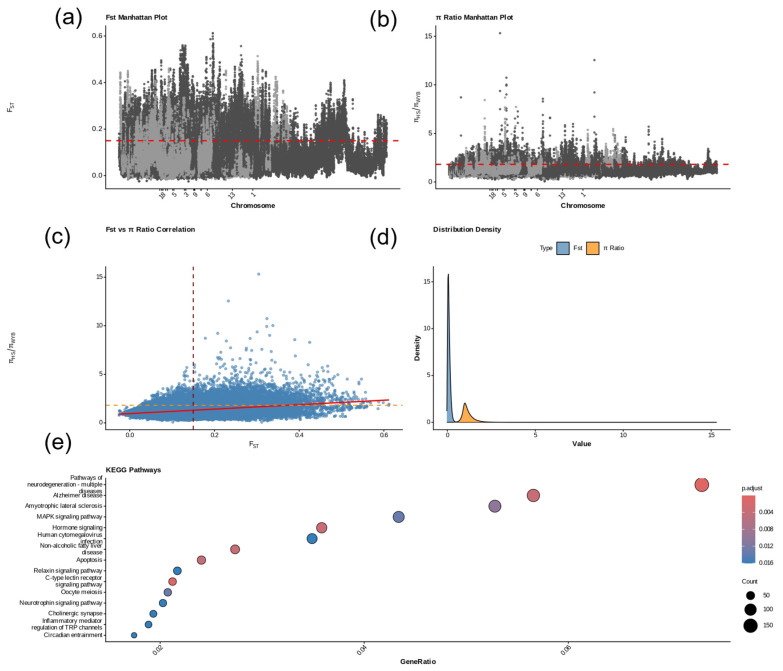
Genomic selection signals and pathway analysis between Wanyue Black pigs (WYB) and Huoshao Black pigs (HS). (**a**) FST Manhattan plot: Genome-wide distribution of FST calculated with a 50 kb sliding window and 25 kb step. Peaks represent regions with significant genetic differentiation between WYB and HS. (**b**) Nucleotide diversity ratio (πHS/πWYB) Manhattan plot: Distribution of πHS/πWYB under the same parameters. Higher ratios indicate genomic regions in WYB with reduced nucleotide diversity due to selective sweeps. (**c**) Correlation plot between FST and nucleotide diversity ratio: Positive correlation between FST and πHS/πWYB supports the reliability of the identified candidate regions. (**d**) Density distribution plot: Density curves of FST and πHS/πWYB values showing the characteristic pattern of “a few genomic regions under strong selection.” (**e**) KEGG enrichment plot: Fifteen significantly enriched pathways of candidate genes. Bubble size represents the number of genes, and color indicates the GeneRatio.

Based on whole-genome resequencing data from 29 Huoshou Black pigs (HS) and 30 Wanyue Black pigs, we employed a sliding window approach (50 kb window with 25 kb step size) to detect genomic regions under selection in WYB relative to HS using a combined analysis of fixation index (F_ST) and nucleotide diversity ratio (π_HS/π_WYB). Windows within the top 5% of F_ST values (>0.15) and simultaneously within the top 5% of π ratios were defined as candidate selective regions, which were subsequently subjected to KEGG pathway enrichment analysis. The results showed that F_ST values ranged from 0.0 to 0.6, with pronounced peaks of genetic differentiation observed on several chromosomes (Figure 6a). In the π ratio Manhattan plot, π_HS/π_WYB values ranged from 0 to 15, and several windows displayed markedly elevated ratios, suggesting selective sweeps in WYB (Figure 6b). Correlation analysis revealed that genomic windows with high F_ST values were frequently associated with elevated π ratios, thereby reinforcing the reliability of the identified candidate selective regions (Figure 6c). Density distribution analysis showed that F_ST values were mainly concentrated in the lower range, whereas π ratios exhibited a multimodal pattern with distinct density peaks in the high-value range—consistent with the characteristic pattern of strong selection acting on a limited number of genomic regions (Figure 6d). KEGG enrichment analysis revealed that the candidate genes were significantly enriched in 15 metabolic and signaling pathways (*p*.adjust < 0.016), including circadian rhythm regulation and the MAPK signaling pathway, which are functionally associated with key biological processes such as growth and development, reproductive regulation, and immune responses (Figure 6e). These findings are highly consistent with the breeding objectives and adaptive characteristics of the Wanyue Black pig during its development and selective breeding history.

## 4. Discussion

This study focuses on the body size traits of the Wanyue Black Pig, aiming to clarify the core value of body size traits as target traits and explore the underlying genetic mechanisms, thereby providing theoretical support for high-quality breeding of Chinese indigenous pig breeds. The Wanyue Black Pig is widely recognized for its tender meat, high reproductive capacity, and rapid growth rate. Body measurements, as key indicators for evaluating pig growth performance, not only serve as the foundation for assessing production performance and growth-related traits but also directly influence the production performance of commercial pigs. They play an indispensable role in determining growth traits.

Based on this, we selected 139 Wanyue Black pigs as the experimental population and performed GWAS. Through functional annotation and phenome-wide association studies, we further investigated significant SNP loci associated with body size traits in pigs. By integrating the candidate genes mapped to these SNP loci with their associations to economic traits, we ultimately identified eight potential candidate genes. Notably, the PheWAS analysis revealed that six genes (*COG6*, *LHFPL6*, *NHLRC3*, *ALG9*, *ATP23* and *RAB28*) showed significant associations with carcass, reproductive, and production traits in pigs. In addition, TWAS indicated that the expression level of the *PTH2R* gene was closely related to pituitary traits, suggesting a potential role in body size regulation and endocrine metabolism. These findings not only enhance our understanding of the genetic regulatory mechanisms underlying body size traits in pigs but may also provide novel molecular markers for future breeding improvement. The following sections will elaborate on the specific functions of these candidate genes and their potential biological significance.

Based on this, a genome-wide association analysis was conducted using a population of 139 Wanyue Black pigs. In total, four SNPs significantly associated with body size traits were identified, and six potential candidate genes—*COG6*, *LHFPL6*, *NHLRC3*, *ALG9*, *ATP23*, and *RAB28*—were further prioritized. In addition, transcriptome-wide association study results revealed that the expression level of PTH2R was significantly associated with pituitary-related traits, suggesting a potential role in body size regulation and endocrine metabolism. Collectively, these findings provide novel insights into the genetic regulatory mechanisms underlying body size traits in pigs and offer valuable candidate genes and molecular markers for future breeding and genetic improvement programs. The functional roles and biological significance of these candidate genes are discussed in detail in the following sections.

*COG6* is a component of the Conserved Oligomeric Golgi (COG) complex, which plays an essential role in maintaining glycosylation and protein trafficking within the Golgi apparatus [28]. Efficiency of *COG6* disrupts Golgi function, resulting in impaired cellular signaling and receptor activity. Studies have shown that patients with *COG6* deficiency present with developmental delay, growth retardation, and microcephaly, suggesting that *COG6* is critical for normal somatic and neural development [29]. Moreover, *COG6* contributes to severe growth impairment through multiple mechanisms, including altered glycosylation, disrupted metabolism, and impaired skin barrier function [30]. These findings suggest that *COG6* may play an important role in animal growth and the determination of body size traits.

*LHFPL6* belongs to the tetraspan transmembrane family and may indirectly regulate growth rate by modulating cell membrane signal transduction and associated metabolic pathways [31]. A probe-based CNV association study identified an 89 kb deletion embedded within the *LHFPL6* gene on bovine chromosome 12, which was significantly associated with average daily gain (ADG). This finding suggests that deletion of *LHFPL6* may influence growth traits in cattle by perturbing growth-related molecular pathways [32]. Therefore, *LHFPL6* may represent an important candidate gene underlying genetic variation in pig body size traits. *NHLRC3* (NLR Family CARD Domain Containing 3) is a member of the NOD-like receptor (NLR) family and encodes a protein involved in immune regulation and cell signaling [33]. A study integrating GWAS with 16S rDNA sequencing of fecal and vaginal microbiota revealed that NHLRC3 and other genes may regulate immune pathways involved in host responses to Brucella infection, thereby influencing animal health and production performance [34]. These findings suggest that *NHLRC3* may indirectly affect body size traits in pigs through immune–metabolic pathways.

*ALG9* encodes alpha-1,2-mannosyltransferase, an enzyme essential for N-linked glycoprotein synthesis in the endoplasmic reticulum [35]. Dysfunction of *ALG9* disrupts proper protein glycosylation, leading to abnormal folding and impaired trafficking of glycoproteins, which can affect cellular growth and signaling pathways [36]. In humans, mutations in *ALG9* are associated with congenital disorders of glycosylation, presenting with growth retardation, skeletal abnormalities, and developmental delay [37]. These findings suggest that *ALG9* may play a pivotal role in regulating growth processes and body size traits in animals through its effects on glycoprotein biosynthesis and associated signaling pathways. *ATP23* encodes a metalloprotease essential for the processing and assembly of the mitochondrial F_1F_0-ATP synthase complex [38]. Impaired *ATP23* function can lead to defective mitochondrial energy production, affecting cellular metabolism and tissue growth. Studies have shown that *ATP23* deficiency in model organisms results in reduced growth and developmental delays due to compromised ATP synthesis and altered metabolic homeostasis [39]. Therefore, *ATP23* may influence body size traits in pigs by modulating energy metabolism and supporting proper cellular growth and tissue development. *RAB28* belongs to the RAB family of small GTPases and is involved in intracellular vesicle trafficking, including endocytosis, exocytosis, and lysosomal transport [40]. Dysregulation of *RAB28* can perturb vesicular transport and signaling, impacting cellular proliferation and tissue growth. Recent studies in model species indicate that RAB28 participates in growth-related pathways, including regulation of nutrient uptake and hormonal signaling [41]. These findings suggest that *RAB28* may contribute to variation in pig body size traits by affecting intracellular transport and associated growth regulatory mechanisms.

PTH2R (Parathyroid Hormone 2 Receptor) is a G-protein-coupled receptor belonging to the class B1 receptor family, which participates in regulating calcium ion transport and other physiological processes [42]. Although studies on PTH2R in relation to mammalian body size are limited, mutations in its homologous receptor PTH1R have been shown to cause delayed maturation of growth plate chondrocytes, shortened tibiae, and impaired weight gain in mice [43]. Previous studies have demonstrated that the TIP39–PTH2R signaling system regulates hypothalamic–pituitary function, influences corticosterone and growth hormone secretion, and may thereby indirectly modulate growth and development through neuroendocrine pathways [44]. Moreover, PTH2R is expressed in the growth plate of neonatal mice and contributes to skeletal development by regulating the proliferation and differentiation of chondrocytes [45]. These findings suggest that PTH2R may influence body size traits via skeletal development and neuroendocrine pathways. In summary, aside from *FOXO1*, the candidate genes identified in this study (*COG6*, *LHFPL6*, *NHLRC3*, *SLCO3A1*, *SV2B*, and *PTH2R*) have been less explored in pigs; however, cross-species evidence indicates that they may contribute to the genetic regulation of body size traits through multiple pathways, including immune modulation, energy metabolism, substance transport, neurotransmitter release, and skeletal development. These insights provide an important theoretical basis for elucidating the molecular mechanisms of body size traits in pigs and for subsequent functional validation studies.

## 5. Conclusions

This study systematically investigated the genetic basis of body size traits in Wanyue Black pigs using high-density SNP genotyping, genotype imputation, and genome-wide association analysis. Following genotype imputation, a total of 4,697,453 high-quality SNPs were obtained, providing a robust foundation for GWAS. The analysis identified four SNPs significantly associated with body length, chest circumference, shank circumference, and leg–hip circumference, and prioritized six candidate genes (*COG6*, *LHFPL6*, *NHLRC3*, *ALG9*, *ATP23*, and *RAB28*). Furthermore, by integrating linkage disequilibrium analysis, transcriptome-wide association studies (TWAS), functional annotation, and phenome-wide association analysis (PheWAS), this multi-dimensional approach provided comprehensive evidence for the functional characterization and potential regulatory mechanisms of the candidate genes. Collectively, these analyses not only reveal the underlying genetic regulatory networks of body size traits but also offer valuable molecular markers for molecular breeding and genetic improvement of Wanyue Black pigs, while laying a foundation for future functional genomics studies.

## Figures and Tables

**Table 1:** Descriptive statistics of body conformation traits in Wanyue Black pigs.

Trait	N	Max	Min	Mean (±SD)	CV
BL	139	89.62	65.38	70.48 ± 9.51	13.49%
CC	139	87.27	62.52	68.57 ± 8.08	11.78%
SC	139	16.14	12.65	14.24 ± 2.03	14.25%
LHC	139	68.54	45.82	58.60 ± 4.96	8.46%

Values are presented as mean ± standard deviation (SD). BL, body length; CC, chest circumference; SC, shank circumference; LHC, leg–hip circumference; CV, coefficient of variation.

**Table 2:** The correlation analysis of physical conformation traits.

Trait	BL	CC	SC	LHC
BL	1	-	-	-
CC	0.956 **	1	-	-
SC	0.914 **	0.935 **	1	-
LHC	0.898 **	0.893 **	0.873 **	1

** indicates significance at *p* < 0.01. BL, body length; CC, chest circumference; SC, shank circumference; LHC, leg–hip circumference.

**Table 3:** Significant SNP Locations for Body Measurements in Wanyue Black Pigs and Annotation of Their Candidate Genes.

Chromosome	SNPID	Position	*p*_wald	Gene
5	rs343622325	23613371	1.528592 × 10^−7^	*ATP23*
8	rs345729102	9521800	2.020554 × 10^−7^	*RAB28*
9	rs343276492	39557329	1.111061 × 10^−7^	*ALG9*
11	rs321308815	14774102	2.042810 × 10^−7^	*LHFPL6*, *GOC6*, *NHLRC3*

## Data Availability

The datasets generated and/or analyzed during the current study are available in the National Genomics Data Center (NGDC), China National Center for Bioinformation (https://ngdc.cncb.ac.cn) under the BioProject accession number PRJCA049068, as of 23 October 2025.
